# Invasion and Amplification of Endogenous Retroviruses in Dasyuridae Marsupial Genomes

**DOI:** 10.1093/molbev/msae160

**Published:** 2024-08-05

**Authors:** Emma F Harding, Lewis K Mercer, Grace J H Yan, Paul D Waters, Peter A White

**Affiliations:** School of Biotechnology and Biomolecular Science, UNSW Sydney, Sydney, Australia; School of Biotechnology and Biomolecular Science, UNSW Sydney, Sydney, Australia; School of Biotechnology and Biomolecular Science, UNSW Sydney, Sydney, Australia; School of Biotechnology and Biomolecular Science, UNSW Sydney, Sydney, Australia; School of Biotechnology and Biomolecular Science, UNSW Sydney, Sydney, Australia

**Keywords:** endogenous virus, virus evolution, marsupial, retrovirus, DFTD, Tasmanian devil

## Abstract

Retroviruses are an ancient viral family that have globally coevolved with vertebrates and impacted their evolution. In Australia, a continent that has been geographically isolated for millions of years, little is known about retroviruses in wildlife, despite the devastating impacts of a retrovirus on endangered koala populations. We therefore sought to identify and characterize Australian retroviruses through reconstruction of endogenous retroviruses from marsupial genomes, in particular the Tasmanian devil due to its high cancer incidence. We screened 19 marsupial genomes and identified over 80,000 endogenous retrovirus fragments which we classified into eight retrovirus clades. The retroviruses were similar to either *Betaretrovirus* (5/8) or *Gammaretrovirus* (3/8) retroviruses, but formed distinct phylogenetic clades compared to extant retroviruses. One of the clades (MEBrv 3) lost an envelope but retained retrotranspositional activity, subsequently amplifying throughout all Dasyuridae genomes. Overall, we provide insights into Australian retrovirus evolution and identify a highly active endogenous retrovirus within Dasyuridae genomes.

## Introduction

Retroviruses are a ubiquitous family of RNA viruses of evolutionary and medical importance. Within this family, there are 11 genera across two subfamilies capable of infecting all vertebrates (Coffin et al. 2021). The unique replication cycle of retroviruses involves reverse transcription of the RNA viral genome and subsequent integration into host genomic DNA, making retroviral infections difficult to clear once established. If this integration occurs in the host's germline, the resulting endogenized retroviral genome can become fixed in a population through drift. Once integrated, endogenous retroviruses (ERVs) lose protein coding capabilities over time and many are epigenetically silenced, however some retain limited RNA transcription and protein expression with largely unexplored consequences (Hurst and Magiorkinis 2015; Gemmell et al. 2016).

ERVs pervade every vertebrate genome studied and comprise 5% to 10% of mammalian genomes (Lander et al. 2001). ERVs often represent unsampled or extinct retroviral lineages, and therefore alternate, broader classification systems are used compared to modern retroviruses. ERVs are classified into three classes based on their polymerase: Class I (*Gammaretrovirus*/*Epsilonretrovirus-*like), Class II (*Alpharetrovirus*/*Betaretrovirus*/*Deltaretrovirus*/*Lentivirus-*like), and Class III (*Spumaretrovirinae-*like) to encompass the broad diversity of ERV sequences (Llorens et al. 2008; Gifford et al. 2018). To add further complexity to ERV classification, they can recombine both pre and post integration to form novel gene combinations (Schwartzberg et al. 1985; Henzy and Johnson 2013; Löber et al. 2018; Yang et al. 2021). Morphological “Type D” retroviruses like Mason-Pfizer monkey virus arose from a recombination event between *Betaretrovirus* and *Gammaretrovirus* in the simian lineage (Sonigo et al. 1986).

Once integrated, ERVs can also interact with other genomic elements, leading to recombination, insertions, and deletions within ERVs. The most common rearrangement is the formation of solo-long terminal repeats (LTRs)—a process wherein the internal coding sequence of the ERV is deleted through homologous recombination, leaving only one long terminal repeat (Hughes and Coffin 2004; Gemmell et al. 2016). Solo-LTRs are estimated to vastly outnumber canonical ERVs in mammalian genomes (Belshaw et al. 2007; Gemmell et al. 2016). If ERVs lose their ability to form virions but retain their intracellular retrotransposition functionality, they transition to a retrotransposon (Smit 1996). Major vertebrate LTR-retrotransposon families share many similarities with retroviruses and are postulated to have arisen from a common ancestor (Smit 1996; Llorens et al. 2008; Koonin et al. 2015). The interwoven evolution of ERVs and retrotransposons combined with their recombination capacity makes the classification of ERVs a challenging endeavor.

The integration and retrotransposition of ERVs have been associated with cellular dysfunction and disease; some exogenous retroviruses like HTLV-1 contain oncogenes that directly cause transformation, whilst others dysregulate tumor suppressor genes or protooncogenes through physical integration or through the addition of promoter regions (Bevilacqua 2022). The expression of proteins from human ERVs is correlated with a myriad of cancers and autoimmune disease (Zhang et al. 2019; Kitsou et al. 2023). Over longer periods of time, the accumulation of ERVs within a genome can lead to genomic instability and dysregulation of regulatory genes (Campbell et al. 2014; Jansz and Faulkner 2021).

Koalas, an iconic marsupial species endemic to Australia, are currently threatened by a retrovirus epidemic which has been correlated with heightened cancer incidence and impaired immune function (McEwen et al. 2021). Koala retrovirus (KoRV) is transmitted both horizontally through infection and vertically through ERVs, with as many as 100 copies per genome in some individuals (McEwen et al. 2021). The extensive genomic invasion can lead to long-term genomic instability, and in vulnerable marsupial populations, this could be detrimental for species survival.

Tasmanian devils, an Australian marsupial in the Dasyuridae family, are afflicted by two transmissible cancers of independent origin: Devil Facial Tumor Disease (DFTD) 1 and 2 which are devastating wild populations, reducing numbers by up to 77% in affected areas (Lazenby et al. 2018). Despite extensive cancer-driven population crashes, little is known about the processes involved in initial DFTD oncogenesis and why Tasmanian devils are particularly susceptible. DFTD is characterized by major karyotypic changes and chromosomal rearrangements, which can be catalyzed by homologous recombination between retroelements (O'Neill et al. 1998; Murchison et al. 2010). Whilst retroviruses do not contribute to DFTD transmission, their potential to contribute to initial oncogenesis through ERV-mediated processes or overall genomic destabilization has not been explored (Murchison et al. 2010).

We aimed to characterize the endogenous retroviral landscape within the Tasmanian devil and determine the extent of ERV invasion and duplication within the genome. To identify ERVs that uniquely invaded the Tasmanian devil, we compared their genovirome to 18 other Australian marsupial species. In particular, we aimed to identify any high prevalence ERV lineages within the Tasmanian devil that were absent in other marsupial species that could contribute to genomic instability. Lastly, we aimed to classify ERV sequences and characterize new genera of Australian retroviruses to better understand the evolutionary history of these viruses.

## Results

### Mapping the Tasmanian Devil Genome

To gain a comprehensive overview of the repeat regions, transposable elements and ERVs within Tasmanian devils and their extent of genomic invasion, we screened the genome using RepeatMasker. A large proportion of marsupial genomes is comprised of repeat regions, namely long and short interspersed nuclear elements (LINEs and SINEs) (Gallus et al. 2015). In the Tasmanian devil, a total of 47.23% of the genome was masked as repeat regions, the majority of which were retroelements (34.5%) comprised of LINEs (28.25%), SINEs (4.64%), and LTR elements (1.61%) (Table 1). A total of 77,446 ERV elements were detected, comprising 1.55% of the Tasmanian devil genome (Table 1). The remaining 0.04% of LTR elements were classified as Metaviridae, a group of retrotransposons related to Retroviridae (Llorens et al. 2020).

**Table 1:** Repeat elements within the Tasmanian devil genome

Repeat type	Total elements	Total bp length	Percentage of genome
ERVs	77,446	47,846,258	1.55
Gene-including ERVs	12,900	22,195,626	0.72
Solo LTRs^a^	64,546	25,650,632	0.83
LINEs	2,696,544	872,071,692	28.25
SINEs	1,188,102	143,292,787	4.64

LINE, long interspersed nuclear element; SINE, short interspersed nuclear element; ERV, endogenous retrovirus; LTR, long terminal repeat.

^a^Approximated by subtracting gene-including ERVs from total predicted ERVs.

**Fig. 1.**
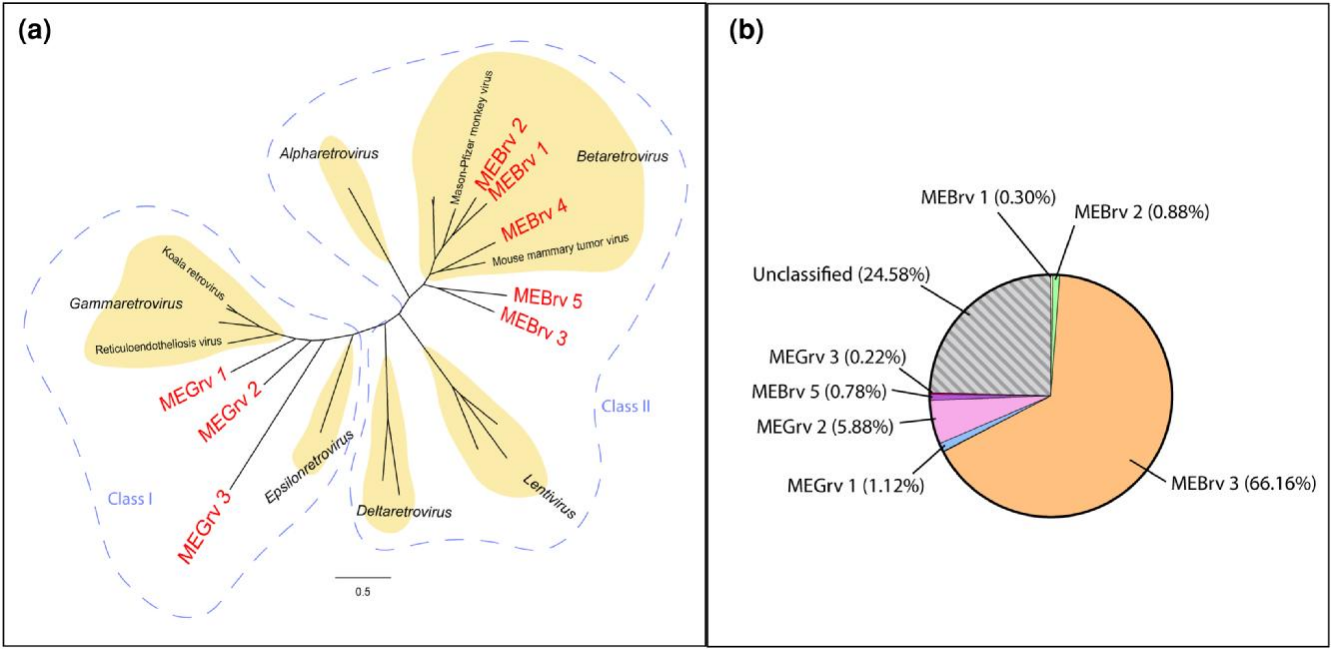
An overview of ERVs in the Tasmanian devil genome. a) Phylogenetic grouping of Tasmanian devil ERVs: 3,500 nt covering the gag/pol regions of representative ERVs > 6 kB in length were aligned using MAFFT. Their phylogeny compared to modern circulating retroviruses was constructed using iqTree2 with 1,000 ultrafast bootstrap replicates. b) Prevalence of each ERV clade within the Tasmanian devil genome. All Tasmanian devil ERVs (*n* = 12,900) were mapped to representative sequences from each of the eight clades for classification. MEBrv 4 is not shown as it comprises <1% (0.08%) of the total ERVs. Unclassified ERVs represent ERVs that are too short or too degraded to be confidently grouped into a clade.

To exclude solo-LTRs and focus on ERVs that have retained internal open reading frames (ORFs), we utilized a custom bioinformatics workflow EVEfinder to detect endogenized retroviral genes (Fig. 1). A total of 12,900 ERVs containing core retroviral genes (gag/pol/env) were identified, comprising approximately 0.7% of the genome (Table 1). These ERVs ranged from short fragments of 68 nt to almost full retrovirus genomes of 9,175 nt, with an average ERV length of 1,720 nt. No full-length proviruses with intact ORFs were detected.

### Classification of Tasmanian Devil ERVs

To classify the 12,900 gene-encoding ERVs in relation to extant retroviruses, we aligned and phylogenetically analyzed all ERVs > 6 kB (*n* = 39). The ERVs grouped into eight clades, five of which were most closely related to modern *Betaretrovirus* (Class II ERVS) and were designated Marsupial Endogenous Betaretroviruses 1-5 (MEBrv 1-5) (Fig. 1a and supplementary fig. S1, Supplementary Material online). The remaining three clades were most closely related to *Gammaretroviruses* (Class I ERVs) and were designated Marsupial Endogenous Gammaretroviruses 1-3 (MEGrv 1-3) (Fig. 1a).

Within *Betaretrovirus*, MEBrv 1 and 2 cluster with Mason-Pfizer monkey virus phylogeny, whilst MEBrv 4 clusters with Mouse mammary tumor virus. Both MEBrv 3 and 5 form a basal phylogeny to extant mammalian betaretroviruses, suggesting that they are marsupial-specific retroviral lineages that do not infect eutherians.

None of the Gammaretrovirus-like marsupial ERVs cluster within modern *Gammaretrovirus*, instead forming branches between *Gammaretrovirus* and *Epsilonretrovirus*, sitting within the broader Class I ERV classification (Fig. 1a). Interestingly, none of the marsupial endogenous gammaretroviruses clustered near koala retrovirus indicating that there is no equivalent lineage infecting Tasmanian devils.

Each representative ERV was compared to previously annotated endogenous retroviruses in the Tasmanian devil to better contextualize their classification (Thibaud-Nissen et al. 2013). The Betaretrovirus MEBrv 1 shared 97.4% nucleotide identity with *Sarcophilus harrisii* endogenous retrovirus group K member 10 (accession: XM_031940153). In addition, the Gammaretrovirus MEGrv 3 shared 86.1% nucleotide identity with *S. harrisii* endogenous retrovirus group S71 (accession: XM_031937583).

To classify the remaining 12,861 shorter ERVs (<6 kB), they were mapped to representatives of the eight ERV clades. This method grouped 9,729 of the total 12,900 ERVs into the eight clades. Interestingly, the majority of these ERVs (*n* = 8,535/66.1%) belonged to MEBrv 3, distantly followed by MEBrv 4 (*n* = 758/5.8%) (Fig. 1b). The other six clades combined comprised only <4% of Tasmanian devil ERVs. The remaining ERVs (*n* = 3,171/24.58%) were unclassifiable due to their degradation, divergence, or short length.

### Identification of Eight Novel Australian Marsupial Retrovirus Clades

**Fig. 2.**
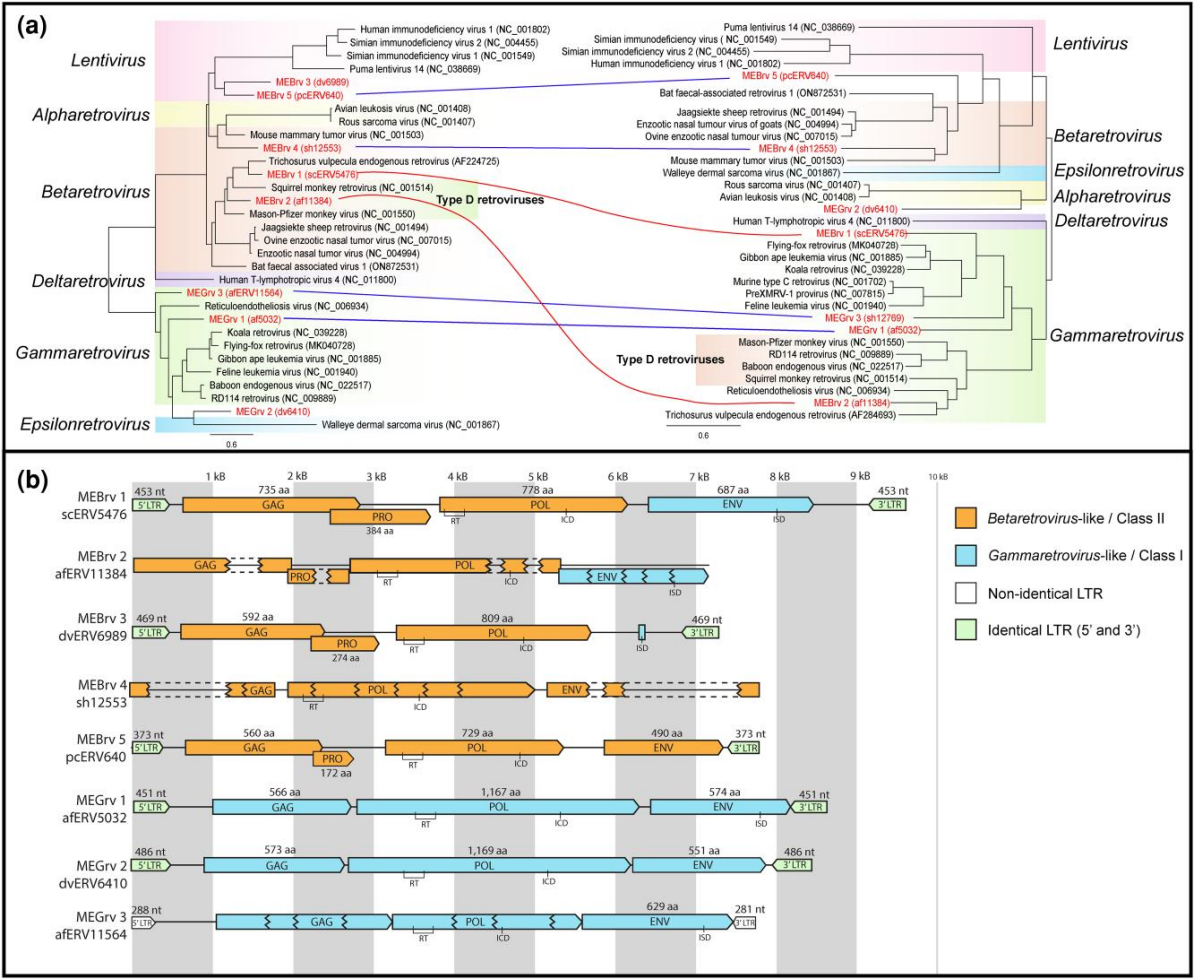
Novel retrovirus lineages. a) Phylogeny of marsupial retrovirus lineages compared to extant genera. The polymerase (pol: 775 nt) and envelope (env: 1,649 nt) ORFs were extracted from endogenous retroviruses and aligned using MAFFT. Phylogenetic trees were constructed using iqTree2 with 1,000 ultrafast bootstraps. Novel marsupial retrovirus clades are denoted with red bold text and recombination is shown with a curved line. A straight line denotes ERVs with no observed recombination. The scale bar represents substitutions per site. b) Genome organization of marsupial retrovirus lineages. Open reading frames were predicted using Geneious Prime and classified using BLASTx searches against the NCBI protein nr database. Discontiguous open reading frames are represented with dashed lines. Conserved motifs are annotated on each sequence: RT, reverse transcriptase; ICD, integrase core domain; ISD, immunosuppressive domain.

Following the grouping of most Tasmanian devil ERVs into eight clades, we expanded our search to include a further 18 Australian marsupial genomes (Table 2). We identified ERVs from these eight clades throughout the Australian marsupials studied, many of which were proviruses with intact ORFs (Fig. 2). These ERVs ranged from 7.3 to 9.5 kB in length with LTRs between 281 and 469 nt (Table 3). Four of the eight clades (MEBrv 1 and 5, MEGrv 1 and 2) have intact ORFs with identical LTRs, indicating that they recently integrated and may still be circulating in Australia (Fig. 2b). One clade (MEBrv 3) was intact aside from the env ORF but has retained intracellular activity (Fig. 2b). The remaining three clades (MEBrv 2 and 4, MEGrv 3) had stop codons interrupting core ORFs, indicating that they are older integrations and unlikely to retain any viral activity (Fig. 2b). The ERVs shared between 32.3% and 55% identity over *gag*, 44.4% and 75.4% identity over *pol*, and 27.3% and 66.5% identity over *env* to characterized retroviruses (Table 3).

**Table 2:** Australian marsupial genomes selected for this study

Genome	Animal	Scientific name	Family	Genome size (Gb)	Scaffolds	Scaffold N50 (Mb)	Genome coverage	ERVs identified
mSarHarr_1.11	Tasmanian devil	*Sarcophilus harrisii*	Dasyuridae	3.1	105	611.3	88	12,900
Sminthopsis_crassicaudata_HiC	Fat-tailed dunnart	*Sminthopsis crassicaudata*	Dasyuridae	3.2	1,644	579.9	…	16,027
USYD_Astu_M	Brown antechinus	*Antechinus stuartii*	Dasyuridae	3.2	487	636.7	77	7,282
mMyrFas1.20211206	Numbat	*Myrmecobius fasciatus*	Myrmecobiidae	3.4	112,292	222.8 kB	31	561
UniMelb_DasViv_v1.0	Quoll	*Dasyurus viverrinus*	Dasyuridae	3.1	76	628.5	30	14,127
ThyCyn2.0_hybrid_assembly	Thylacine	*Thylacinus cynocephalus*	Thylacinidae	3.4	149,109	629.1	50	3,047
AdamAnt	Yellow-footed antechinus	*Antechinus flavipes*	Dasyuridae	3.2	487	636.7	100	19,399
mMacEug1.pri	Tammar wallaby	*Notamacropus eugenii*	Macropodidae	3.4	314	489.7	30.4	4,765
Lagorchestes_hirsutus_HiC	Rufous hare-wallaby	*Lagorchestes hirsutus*	Macropodidae	3.4	569,642	401.3	93	2,359
mf-2k	Western grey kangaroo	*Macropus fuliginosus*	Macropodidae	3.6	1,417,799	341.3	64	1,725
mg-2k	Eastern grey kangaroo	*Macropus giganteus*	Macropodidae	3.5	1,012,328	392.9	70	2,333
LBP_v1	Leadbeater's possum	*Gymnobelideus leadbeateri*	Petauridae	3.5	12,502	502.5	100	352
PetGlider_PUasm1.0	Sugar glider	*Petaurus breviceps papuanus*	Petauridae	3.5	1,895	28.2	71	2,835
mTriVul1.pri	Brushtail possum	*Trichosurus vulpecula*	Phalangeridae	3.4	211	446.2	60.7	5,472
Pseudocheirus_occidentalis_HiC	Western ringtail possum	*Pseudocheirus occidentalis*	Phalangeridae	3.6	917,762	213.5	60	2,587
phaCin_unsw_v4.1	Koala	*Phascolarctos cinereus*	Phascolarctidae	3.2	…	…	57.3	715
mBetpen1.pri.20210916	Woylie	*Bettongia penicillata ogilbyi*	Potoroidae	3.4	1,116	6.9	76.9	726
Potorous_gilbertii_HiC	Gilbert's potoroo	*Potorous gilbertii*	Potoroidae	3.8	1,148,168	557.8	71	2,434
bare-nosed wombat genome assembly	Wombat	*Vombatus ursinus*	Vombatidae	3.5	15,415	28.5	87	1,136

**Table 3:** Novel retrovirus clades detected in Australian marsupial genomes

Clade	Length (kB)	Representative	Potentially active	GAG (% identity)	POL (% identity)	ENV (% identity)	LTR length	Extant genera	Predicted morphology	ERV class
MEBrv 1	9.5	scERV5476	Yes	*Trichosurus vulpecula* endogenous retrovirus (55%)	*Trichosurus vulpecula* endogenous retrovirus (75.4%)	Murine leukemia virus (37.6%)	453	*Betaretrovirus*	Type D	II
MEBrv 2	>7	afERV11384	No	Simian retrovirus 2 (45.9%)	Simian retrovirus 8 (53.0%)	Squirrel monkey retrovirus (66.5%)	…	*Betaretrovirus*	TypeD	II
MEBrv 3	7.3	dvERV6989	Yes^a^	Cervid endogenous Betaretrovirus 1 (26.0%)	Bat fecal associated retrovirus 1 (44.4%)	…	469	*Betaretrovirus*	Type A (IAP)	II
MEBrv 4	>9.2	sh12533	No	Mouse mammary tumor virus (40.0%)	Mouse mammary tumor virus (47.3%)	Bat fecal associated retrovirus 1 (29.2%)	…	*Betaretrovirus*	Type B	II
MEBrv 5	7.8	pcERV640	Yes	Jaagsiekte sheep retrovirus (32.3%)	Simian retrovirus 4 (49.0%)	Bat fecal associated retrovirus 1 (27.3%)	373	*Betaretrovirus*	Type B	II
MEGrv 1	8.5	afERV5032	Yes	Duck infectious anemia virus (52.9%)	Reticuloendotheliosis virus (55.9%)	Mouse endogenous murine leukemia virus (39.7%)	451	*Gammaretrovirus*	Type C	I
MEGrv 2	8.5	dv6410	No	*Rhinolophus ferrumequinum* retrovirus (27.3%)	Reticuloendotheliosis virus (45.3%)	Reticuloendotheliosis virus (28.6%)	486	*Gammaretrovirus*	Type C	I
MEGrv 3	7.8	afERV11564	No	Reticuloendotheliosis virus (53.0%)	Xenotrophic MuLV-related virus (53.7%)	Feline leukemia virus (33.2%)	281	*Gammaretrovirus*	Type C	I

^a^Unable to form extracellular virions.

Phylogenetic trees of the *gag*, *pol*, and *env* were constructed to identify evidence of recombination events and provide insight into the evolutionary history of marsupial ERVs (Fig. 2 and supplementary fig. S2, Supplementary Material online). The polymerase was used to classify each ERV clade as either Class I, Class II, or Class III. MEBrv 1, 2, 3, 4, and 5 polymerase regions clustered within modern *Betaretrovirus* representatives and can thus be categorized as Class II ERVs (Fig. 2 and Table 3). MEGrv 1, 2, and 3 polymerase regions clustered with modern *Gammaretrovirus* and *Epsilonretrovirus* and were subsequently classified as Class I ERVs (Fig. 2 and Table 3).

Viruses from four of the eight marsupial ERV clades shared identity over one or more core regions (*gag*/*pol*/*env*) with beta- and gammaretroviruses from other Australian mammals, including the brushtail possum (*Trichosurus vulpecula* endogenous retrovirus), grey-headed flying fox (bat fecal associated retrovirus 1), black flying fox (flying-fox retrovirus), and an introduced deer (cervid endogenous betaretrovirus 1) (Figs. 1a and 2a and Table 3). The other four ERV clades were not closely related to previously identified Australian retroviruses, indicating that they may be recent introductions into Australia rather than coevolved Australian lineages of older retroviruses.

Within the *Betaretrovirus* genus, viruses are further classified as either Type-B or Type-D based on the origin of their envelope and subsequent morphology: Type-B betaretroviruses contain a betaretrovirus envelope whilst Type-D contain a gammaretrovirus envelope (Coffin et al. 2021). Within this study, we identified five ERVs with identity to betaretroviruses, three of which were Type-B and two of which were Type-D (Table 3).

The three Type-B betaretrovirus lineages (MEBrv 3, 4, and 5) formed a sister clade to modern Type-B mammalian betaretroviruses; a group including mouse mammary tumor virus and jaagsiekte sheep retrovirus (Figs. 1a and 2a and Table 3). They shared between 26% and 49% nt identity over core regions (*gag*/*pol*/*env*) to modern Type-B retroviruses (Table 3), suggesting an ancient divergence of these Australian ERVs from the Type-B betaretroviruses of other continents. The *env* had the lowest identity to previously characterized betaretroviruses, sharing only 27% to 29% nt identity with their nearest relative, bat fecal associated virus 1.

The other two betaretrovirus lineages (MEBrv 1 and 2) clustered with modern Type-D betaretroviruses including Mason-Pfizer monkey retrovirus and *T. vulpecula* retrovirus (Figs. 1a and 2a and Table 3). They shared between 37% and 66% identity over core regions (*gag*/*pol*/*env*) to non-Australian Type-D betaretroviruses (Table 3), again suggesting an ancient divergence between Australian ERVs and those of other continents.

Like their phylogenetic relatives, the two Type-D lineages were also recombinant viruses with betaretrovirus *gag*/*pol* regions and gammaretrovirus *env* regions (Fig. 2). Interestingly, although they both have *env* regions with identity to gammaretrovirus, they fall into two distinct phylogenetic clusters: MEBrv 1 clusters with Type-C gammaretroviruses like feline leukemia virus and gibbon ape leukemia virus, whilst MEBrv 2 clusters with Type-D betaretroviruses like Mason-Pfizer monkey retrovirus and *T. vulpecula* retrovirus (Fig. 2a and Table 3). This suggests that at least two distinct envelope recombination events lead to the rise of these two marsupial ERV clades.

The three Gammaretrovirus lineages (MEGrv 1, 2, and 3) shared identity with both avian (reticuloendotheliosis virus and duck infectious anemia virus, 29% to 56%) and also mammalian retroviruses (murine leukemia virus, feline leukemia virus, and *Rhinolophus ferrumequinum* retrovirus, 27% to 40%) over the core regions (*gag*/*pol*/*env*) (Figs. 1a and 2a and Table 3).

### Dasyuridae-Family Marsupials Are Enriched for ERVs

In the Tasmanian devil, we observed a pattern of Class I and Class II ERVs from eight major clades, of which one (MEBrv 3) predominated (Fig. 1b). To determine if similar patterns pervaded other Australian marsupials, we expanded our ERV search to include a further 18 Australian genomes from seven marsupial families (Table 2). We also included three genomes from American marsupials: the South American agile grace opossum and gray short-tailed opossum, and the North American Virginia opossum (supplementary table S1, Supplementary Material online).

**Fig. 3.**
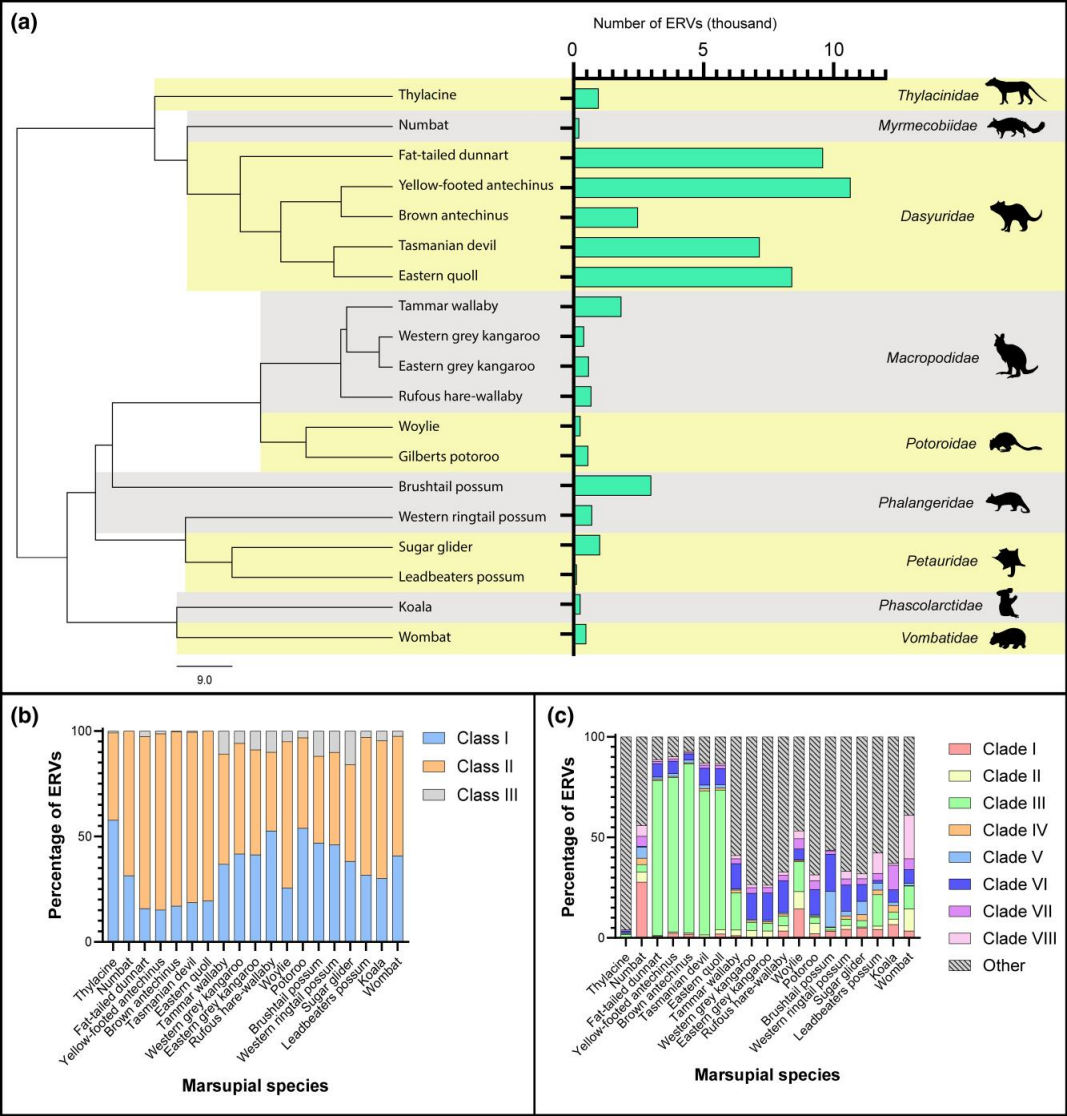
An overview of ERV prevalence and classification in Australian marsupials. a) Correlation of marsupial phylogeny and number of ERVs. Marsupial families are alternately shaded. Scale bar represents million years. The marsupial host tree was generated at TimeTree.org (Kumar et al. 2017). b) Classification of marsupial ERVs into endogenous classes. Classification was based on their closest relatives as determined by a tBLASTn search. c) Prevalence of each Tasmanian devil ERV clade within Australian marsupial genomes. ERVs were classified using the classify sequences tool in Geneious Prime using representative sequences from each clade.

In the 18 additional Australian species, a total of 87,882 ERVs containing core retroviral genes were identified. Surprisingly, all of the Dasyuridae marsupials had many more ERVs (∼10×) compared to other marsupial species (Fig. 3a). Within the Dasyuridae, the yellow-footed antechinus had the most ERVs (*n* = 19,399, average length = 1,757), followed by the fat-tailed dunnart (*n* = 16,027, average length = 1,675) and the Eastern quoll (*n* = 14,127, average length = 1,844) (Fig. 3a). In contrast, non-dasyurid marsupials contained significantly less ERVs, ranging from 352 in the Leadbeater’s possum (Family: Petauridae) to 5,472 in the brushtail possum (Family: Phalangeridae) (Fig. 3a).

Within the three American marsupials, both the South American species (gray short-tailed opossum and agile grace opossum) had 18,820 and 9,194 ERVs ranging in size from 68 to 12,196 nt which comprised 0.96% and 0.34% of their genomes, respectively (supplementary table S1, Supplementary Material online). In contrast, the North American Virginia opossum contained only 1,725 ERVs ranging between 68 and 6,668 nt and comprising 0.07% of its genome (supplementary table S1, Supplementary Material online).

All 87,882 Australian ERVs were classified into ERV classes based on their closest relative in the BLASTx search (Fig. 3b). Similar to Tasmanian devils, all marsupial ERVs were primarily *Betaretrovirus-*like (Class II) or *Gammaretrovirus-*like (Class I) (Fig. 3b).

### Marsupial Endogenous Betaretrovirus 3 ERVs Have Proliferated Throughout Dasyuridae Genomes

To understand whether the eight Tasmanian devil ERV clades were also present in other marsupials, we mapped each ERV to these eight clades. Within all five Dasyuridae species, MEBrv 3 ERVs were overrepresented, comprising an average of 67.5% of all ERVs, and up to 83.0% of ERVs in the brown antechinus (Fig. 3c). In comparison, MEBrv 3 ERVs only comprised between 1.9% and 8.7% of ERVs in the eight other marsupial families (Fig. 3c). Outside of Dasyuridae, most marsupial ERVs did not classify into the eight Tasmanian devil clades, indicating that the ERV composition greatly differs between marsupial families (Fig. 3c).

### MEBrv 3 ERVs Have Lost Their Envelope But Maintained Retrotransposon Activity

**Fig. 4.**
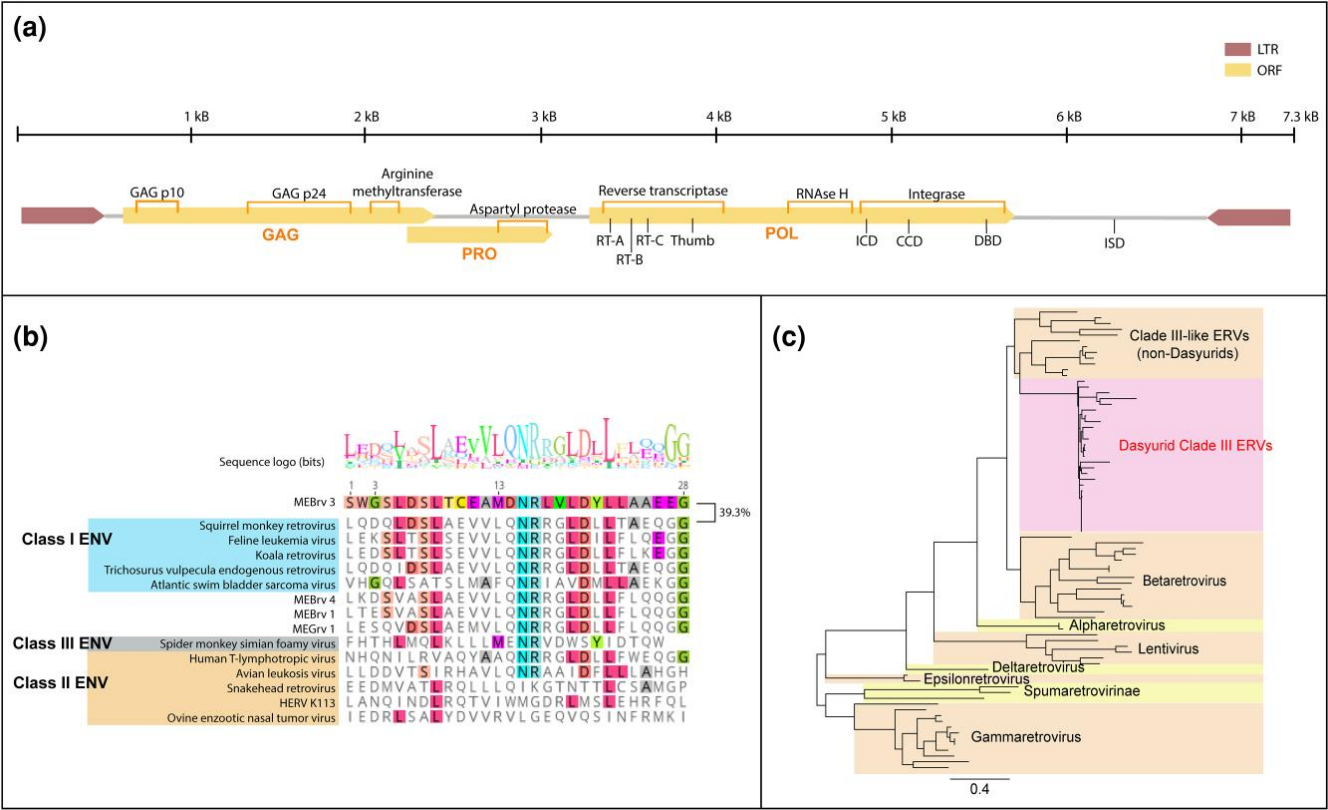
Genome and phylogeny of MEBrv 3 ERVs. a) Genome organization of a representative MEBrv 3. Motifs are labeled and correspond to: RT-A, B, C, reverse transcriptase; ICD, integrase core domain; CCD, catalytic core domain; DBD, DNA-binding domain; ISD, immunosuppresive domain. b) Alignment of immunosuppressive domain of Env proteins. The ISD was identified from each ERV clade (where an env was present) based on the presence of conserved motifs and alignment with extant retroviruses. Domains were aligned using MAFFT and a sequence logo was constructed using Geneious Prime. c) Phylogeny of the MEBrv 3 reverse transcriptase domain. The reverse transcriptase domain of marsupial MEBrv 3 ERVs were translated in silico (174 aa) and aligned with exogenous retroviruses using MAFFT. Phylogeny was determined using iqTree2 with 1,000 ultrafast bootstrap replicates.

MEBrv 3 ERVs comprised the majority of ERVs within all Dasyuridae marsupial genomes. Within these animals, MEBrv 3 ERVs with intact LTRs, *gag*, *pro*, and *pol* ORFs were identified in the Eastern quoll, fat-tailed dunnart, and yellow-footed antechinus genomes. These ERVs were ∼7.3 kB long and contain *gag*, *pro*, and *pol* ORFs, but lack an *env* ORF (Fig. 4a). The ERVs are flanked by two identical LTRs, indicating recent and probably ongoing retrotransposition within the three genomes (Fig. 4a). In contrast, Tasmanian devil MEBrv 3 ERVs were degraded and nonfunctional, with the most intact representative (shERV12898) having interrupted ORFs and LTRs with only 97.5% identity.

An immunosuppressive domain (ISD) was identified beyond the 3′ terminus of the pol ORF, suggesting the presence of a degraded *env* (Fig. 4a). The in silico translated ISD had highest homology with *Gammaretrovirus* and *Epsilonretrovirus* ISD sequences, indicating that a Class I envelope was once present (Fig. 4b). MEBrv 3 ISD was most similar to squirrel monkey retrovirus and feline leukemia virus (39.3% over 28 aa), both of which have Class I *Gammaretrovirus* envelopes. Interestingly, the MEBrv 3 ISD shared little homology (<32.1% over 28 aa) with the other marsupial ERV ISD regions, indicating that the MEBrv 3 envelope was likely from a distinct recombination event rather than divergent evolution.

To investigate whether MEBrv 3 ERVs had retained retrotransposon activity, we constructed a phylogeny of the reverse transcriptase (RT) domain (Fig. 4c). The RT domain of MEBrv 3 ERVs share identity with Class II *Betaretrovirus* sequences but form a sister phylogeny (Fig. 4c). MEBrv 3 Dasyuridae RTs showed rapid sequence divergence from a common ancestor, suggesting intracellular retrotransposition rather than repeated exogenous infection and integration (Fig. 4c).

## Discussion

Our study provides a comprehensive overview of ERVs within Australian marsupial genomes, with a particular focus on Tasmanian devils. The ERVs clustered into eight novel retrovirus lineages which are related to modern *Betaretrovirus* and *Gammaretrovirus* genera (Figs. 1a and 2a). Of these, we identify an ERV lineage (MEBrv 3) that has invaded and amplified throughout the genomes of Australian Dasyuridae marsupials (Figs. 3c and 4).

### Australian Marsupial Retrovirus Diversity

We surveyed 19 Australian marsupial genomes for the presence of endogenized retroviruses to identify novel retroviral lineages (Table 2). Eight clades were identified in the Tasmanian devil genome and used to explore the retroviral landscape in other Australian marsupials.

The eight ERV clades were closest related to modern *Betaretrovirus* and *Gammaretrovirus* viruses, sharing between 26.0% and 75.4% identity to previously characterized viruses (Fig. 2a and Table 3). Representatives or close relatives of these eight clades were identified in all marsupials (Fig. 3c), suggesting widespread prevalence of these retroviruses throughout Australian marsupials. In addition, viruses from four of the eight ERV clades we identified are related to Australian retroviruses in bats (Fig. 2a), suggesting that bats play an integral role in the circulation of retroviruses within Australian fauna. Indeed, bats’ nomadic and migratory lifestyle allows them to spread retroviruses inter- and intra-continentally as demonstrated by their implication for the introduction of koala retrovirus to Australia (Breed et al. 2010; Hayward, Tachedjian, et al. 2013; McMichael et al. 2019; Hayward et al. 2020). Screening Australian bat genomes for the marsupial ERV clades as described herein will further elucidate the role of bats in retrovirus cross-species transmission and evolution in Australia. Sampling of other animals known to be involved in retroviral spread intra-continentally, for example rats, would also be beneficial.

The other four ERV clades did not share high identity with previously characterized Australian retroviruses, instead forming phylogenetic sister clades to retroviruses from other continents (Fig. 2 [top panel] and Table 2). One explanation for their low identity to characterized retroviruses is viruses from the four ERV clades coevolved within Australia since its split from Gondwana, approximately 130 million years ago (Veevers and McElhinny 1976), to form divergent Australian lineages. The betaretrovirus lineages MEBrv 3, 4, and 5 form the representatives of an “Australian” phylogeny, with the closest known relative being Type-B retroviruses like mouse mammary tumor virus (Fig. 2 and Table 3). Sampling of endogenous and exogenous retroviruses in both native and introduced rodents amongst other mammals in Australia will help identify the likely source and evolutionary history of these Australian phylogenies. Additionally, sampling across Indonesia and New Guinea will help elucidate the role of faunal exchange in the introduction and spread of retroviruses within Australia.

The Gammaretrovirus lineages MEGrv1 and MEGrv 3 clades grouped with retroviruses of birds, namely reticuloendotheliosis virus and duck infectious anemia virus (Fig. 2a and Table 3). Host-switching of gammaretroviruses between birds and mammals has been proposed before (Hayward, Grabherr, et al. 2013; Niewiadomska and Gifford 2013), and our findings suggest that similar transitional events may have occurred to give rise to these two retrovirus clades. Further sampling of Australian birds and mammals will help elucidate the origins of these retroviruses.

From the 19 Australian marsupial genomes, we were able to identify fully intact ERVs from five of the eight novel ERV clades, including the identical LTRs (Fig. 2b). This implies that these ERVs are recently integrated, and their exogenous relatives may still be circulating in modern marsupial populations.

### An Ancient Beta–Gamma Recombination Event

To investigate recombination events within Australian retroviruses, phylogenies of the three core retroviral regions (*gag*/*pol*/*env*) were constructed and compared (Fig. 2a). Three of the eight novel ERVs are recombinant viruses between *Betaretrovirus-*like *gag*/*pol* and *Gammaretrovirus-*like *env* (Fig. 2). The differing phylogenies of the *env* within *Gammaretrovirus* support multiple recombination events forming these clades, rather than one recombination event which divergently evolved (Fig. 2a). The envelope of MEBrv 2 ERVs clusters in a phylogeny with TvERV from brushtail possums and reticuloendotheliosis virus in a sister lineage to Type-D retroviruses. In contrast, the envelope of MEBrv 1 ERVs cluster with viruses from Indonesian primates and Australian bats, and, similar to KoRV, was likely imported in through faunal migration rather than evolving in Australia (Hayward et al. 2020). Interestingly, the phylogeny of TvERV from brushtail possums also shows evidence of recombination events: the *gag*/*pol* encoding regions share identity to MEBrv 1 ERVs, yet the *env* encoding region shares identity to MEBrv 2 ERVs (Fig. 2a). The differing *gag*/*pol* and *env* topologies for MEBrv 1, MEBrv 2, and TvERV retroviruses suggest that extensive recombination has occurred between betaretroviruses and gammaretrovirus within Australia.

The prevalence of *Gammaretroviral* envelopes (6/8 clades, Fig. 2b) endogenized within marsupials suggests a selective advantage for this envelope, either through increasing the probability of integration or benefitting viral fitness and/or host range. Throughout mammals and birds, the acquisition of a *Gammaretroviral* envelope by Class II retroviruses has been well documented and is postulated to have given rise to the *Deltaretrovirus* and *Alpharetrovirus* genera (Henzy and Johnson 2013). We show that this overrepresentation of *Gammaretroviral* envelope genes within ERVs is also widely present in Australian marsupials.

### Dasyuridae Have More ERVs Than Other Australian Marsupials Studied

This study screened 19 Australian marsupial genomes to identify ERV prevalence. Amongst species, the number of ERVs identified ranged from 352 in the Leadbeater's possum to 19,399 in the yellow-footed antechinus (Fig. 3a and supplementary table S1, Supplementary Material online). Notably, all members of the Dasyuridae family of carnivorous marsupials studied contained increased ERV prevalence compared to the other Australian marsupial families, with ERVs comprising an average of 0.72% of Dasyuridae genomes (Fig. 3a and supplementary table S1, Supplementary Material online). In contrast, the ERVs in other Australian marsupial families comprised between 0.021% and 0.19% of their genomes (supplementary table S1, Supplementary Material online). These findings are consistent with previous work that identifies more ERVs within the Tasmanian devil genome compared to herbivorous Australian marsupials (Hayward, Grabherr, et al. 2013; Hayward et al. 2015).

Within the Dasyuridae family, we identified increased ERV integrations compared to the other marsupials. These heightened numbers could arise from either more frequent exogenous infection and germline integration, intracellular retrotransposition post-infection, or a combination of both. Due to their carnivorous nature, Dasyuridae are expected to contract more viruses than herbivores due to their close and regular contact with other species (Hayward, Grabherr, et al. 2013). Predation provides ample opportunities for close contact of blood between predator and prey and is postulated to have caused host switching of human immunodeficiency virus and its simian precursors (Bailes et al. 2003; Sharp and Hahn 2011). The Dasyuridae diet primarily consists of small marsupials, mammals, birds, lizards, and insects, providing a large pool for retroviral recombination opportunities (Stannard 2012).

### Biogeographical Isolation Influences ERV Formation and Preservation

To better understand and compare the ERV landscape of marsupials, we screened for the presence of ERVs in 19 Australian and three American marsupials. It has previously been reported that gray short-tailed opossums (*Monodelphis domestica*) have a high prevalence of ERVs compared to other mammals, comprising over 7% of their genome (Hayward, Grabherr, et al. 2013). Concomitant with this, we also found that the gray short-tailed opossum had increased levels of ERVs compared to non-dasyurid Australian marsupials, as did the agile gracile mouse opossum (supplementary table S1, Supplementary Material online). Their ERV prevalence were roughly comparable to that of the Tasmanian devil and other members of Australian Dasyuridae (supplementary table S1, Supplementary Material online).

We speculate that biogeographical process played a major role in the formation and retention of ERVs within the Tasmanian devil population as it became isolated from mainland Australian populations 12,000 years ago (Brüniche-Olsen et al. 2018). Whilst the Tasmanian devil still retains many ERVs (12,900), none of these ERVs were found intact as a provirus, indicating that they are older integrations. In contrast, *dasyurid* marsupials on mainland Australia have many intact ERVs with identical LTRs; hallmarks of recent ERV formation (Fig. 2). We hypothesize that retrovirus diversity is greatly decreased in Tasmania through marsupial population bottlenecks and subsequent viral extinction, leading to no new ERV integration in recent history. As more marsupial genomes become available, comparing ERVs within other Tasmanian marsupials like the pademelon will provide insight into whether this ERV degradation is geographically driven or resulting from unknown genomic factors.

### MEBrv 3 ERV Invasion Could Contribute to Heightened Oncogenesis Frequency in Dasyuridae

The intracellular regulation of ERVs once integrated is primarily via increased DNA methylation, which transcriptionally silences them (Jansz 2019). Undermethylation can release ERVs and other retrotransposons from silencing, as observed for an endogenous retrovirus that amplified throughout an undermethylated wallaby genome (O'Neill et al. 1998). We observed an accumulation of MEBrv 3 ERV copies within Dasyuridae (Fig. 3c), indicating that these elements are unsuccessfully regulated by methylation. In contrast to the tammar wallaby and American opossum, Tasmanian devils have reduced methylation on autosomes, which MEBrv 3 ERV copies may influence within Dasyuridae (Ingles and Deakin 2015).

A main driver of oncogenesis is genomic instability, often caused by the accumulation of mutations, microsatellite instability, or chromosomal recombination (Yao and Dai 2014). Chromosomal recombination is often associated with repeats with a high degree of sequence similarity, like ERV copies, which can misalign during cell division and lead to significant karyotypic changes (Gu et al. 2008). DFTD in Tasmanian devils is associated with extensive chromosomal rearrangements resulting in distinct tumor karyotypes (O'Neill et al. 1998; Ingles and Deakin 2015).

MEBrv 3 ERV amplification is evident across Dasyuridae (Fig. 3c) and could contribute to the genomic instability required for oncogenic chromosomal rearrangements. MEBrv 3 ERVs lack an envelope gene yet retain retrotransposon activity (Fig. 4a and c). The loss or degradation of env genes is associated with increased proliferation throughout a genome, an example of which is the intracisternal A-type particles (IAPs) in mice: defective ERVs which are present in up to 1,000 copies in the mouse genome (Dupressoir and Heidmann 1997; Qin et al. 2010; Magiorkinis et al. 2012). MEBrv 3 ERVs comprised an average of 67.5% of all ERVs within Dasyuridae, suggesting that they proliferated through a similar mechanism to IAPs after envelope loss.

IAPs are expressed in a range of tumors of mice including leukemia, mammary tumors, epidermal carcinoma, melanoma, and neuroblastoma (Hojman and Périès 1986; Li et al. 1996) and are potent instigators of de novo germline mutations (Maksakova et al. 2006). IAP expression is also inversely correlated with the expression of major histocompatibility complex (MHC) Class I H-2K^b^ in melanoma in mice (Li et al. 1996). This protein complex has fewer alleles in Tasmanian devil populations compared to other marsupials and eutherian mammals and is not expressed in DFTD cells (Caldwell and Siddle 2017). The decreased diversity of MHC Class I molecules in Tasmanian devils and abundance of IAP-like Class III ERVs could provide an optimal environment for the spontaneous development of cancers.

Aside from the Tasmanian devil, other Dasyuridae marsupials have been documented with unusual spontaneous neoplasia including quolls, dunnarts, and antechinus (Attwood and Woolley 1973). The kowari (*Dasyuroides byrnie*) is predicted to have the highest cancer mortality rate of any mammal, at 57.14% (Vincze et al. 2022). The proliferation of MEBrv 3 ERVs provides a possible explanation for the increased cancer incidence within Dasyuridae marsupials. Further research investigating the ERV landscape within DFTD tumors and cells would provide insight into their role in initiating oncogenesis.

## Materials and Methods

### Initial Screen of Tasmanian Devil Genome for Repeat Regions

An initial screen was undertaken to estimate the abundance of repeat elements, transposons, and ERVs within the Tasmanian devil genome. To perform de novo identification of repeat families, the genome was screened with RepeatModeler2 (Flynn et al. 2020). The resulting consensus repeat sequences were used as the repeat database for RepeatMasker (http://www.repeatmasker.org) to estimate the transposable element composition of the Tasmanian devil genome. Calculation of solo-LTR abundance was done by subtracting the gene-containing ERVs (below) from the total LTR elements detected by RepeatMasker.

### Identification of Gene-Containing ERVs in the Tasmanian Devil Genome

**Fig. 5.**
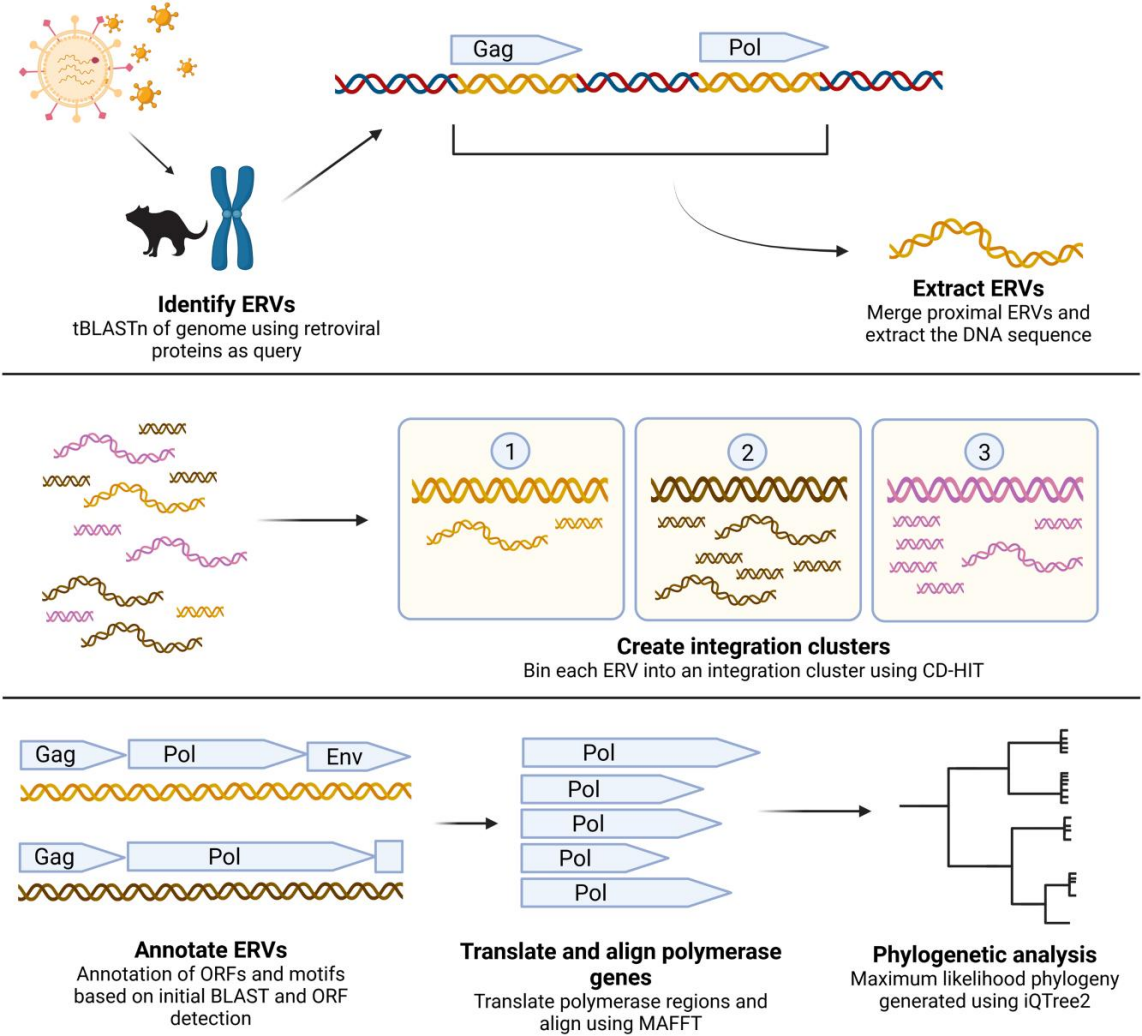
Overview of the custom bioinformatics workflow used for this study. ERVs are identified using a tBLASTn search against representative marsupial genomes. Resulting hits are binned into integration clusters based on their percentage identity. Representatives from each integration cluster are annotated and the polymerase region is used for phylogenetic analysis.

A custom BLAST-based workflow, EVEfinder, was developed to detect EVEs from any viruses in any host genome, given an input database of target viral proteins (Fig. 5). In this study, we used 486 retroviral proteins representing the taxonomical diversity of retroviruses to identify host genomic regions with similarity (when in silico translated) to retroviral core regions (*gag*/*pol*/*env*). This methodology specifically excluded solo-LTRs and instead focused on ERVs that retained partial or full protein coding regions. Hits from the initial tBLASTn searches within 1,000 nt proximity were merged to create ERV genomic sequences. ORFs present in each ERV and their predicted genera were designated based on their closest related virus in the initial tBLASTn search. The DNA sequence of each ERV was extracted and numbered based on host chromosomal position.

### Determining the Copy Number of Each ERV

To estimate the copy number of each unique ERV within a genome, a clustering algorithm was used to sort closely related ERVs into integration clusters based on NT identity (>80%) using cdHit-est (v4.8.1) (Li and Godzik 2006) (word size = 4). The longest member of each cluster was designated the representative cluster ERV sequence and manually curated as described previously (Goubert et al. 2022).

### Classification of ERVs and Identification of Novel Retroviral Genera in the Tasmanian Devil

To group ERVs into lineages and predict their closest modern genera, phylogenetic analysis was conducted on each integration cluster > 6 kB in the Tasmanian devil. The initial lineage classification was based on the polymerase region: nucleotide sequences covering the polymerase (∼2 kB) were aligned using MAFFT 7.481 (Katoh and Standley 2013) and a phylogenetic tree was constructed using iqTree2 with 1,000 ultrafast bootstrap replicates (Minh et al. 2020). Using this clustering, a representative ERV from each lineage was selected based on the sequence length and integrity of ORFs as predicted by Geneious Prime 2023.2.1 (https://www.geneious.com).

To compare Tasmanian devil ERVs to previously characterized ones, MEGABLAST searches (*e*-value 1e^−03^) were conducted using annotated Tasmanian devil ERVs from the automated genome annotation (*S. harrisii* annotation release 103) and pan-Mars-Env2 (accession: KM235347).

To subsequently classify all shorter ERVs in the Tasmanian devil, they were mapped to each representative sequence above. Mapping was undertaken using the “Classify Sequences” tool in Geneious Prime (Very High Sensitivity, minimum overlap 50 bp) to classify each of the ERVs into one of the representative lineages identified through phylogenetic analysis.

ERVs were named as either MEBrv (Marsupial endogenous betaretrovirus) or MEGrv (Marsupial endogenous gammaretrovirus) based on their relations to modern retrovirus genera.

### Classification of ERVs Throughout Marsupial Genomes

To determine if ERV lineages present in the Tasmanian devil extended to other marsupial species, the custom EVEfinder pipeline was run on each of the other 18 Australian marsupial genomes (Table 2). The resulting EVEs were classified into one of the eight Tasmanian devil EVE lineages using the “Classify Sequences” tool in Geneious Prime (as above) or classified as “other” if they did not have a hit.

### Selection and Annotation of Representative ERVs From Each Novel Lineage

For each novel ERV lineage, the longest and most intact ERV (retaining the most ORFs) identified from all 19 marsupial genomes was selected as the clade representative. Eight sequences from five marsupial genomes were chosen to represent the eight clades (Table 3).

These representative sequences were annotated based on the presence of open reading frames (“Find ORF” tool in Geneious Prime) and *gag*, *pol*, and *env* motifs. Predicted ORFs were confirmed based on alignments with related extant retroviruses and the presence of in-frame enzyme motifs. For intact ERVs, LTR regions were identified using the “Find Repeats” function in Geneious Prime with a minimum repeat length of 100% and 20% maximum mismatches. LTRs were confirmed by aligning 2 kB upstream and downstream of the annotated ORFs.

The *gag*, *pol*, and *env* nucleotide regions of each clade representative were phylogenetically analyzed as above.

## Supplementary Material

msae160_Supplementary_Data

## Data Availability

The custom workflow developed for this study (EVEfinder) is available on GitHub (https://github.com/emma-harding/EVEfinder). This repository contains the retrovirus protein database used to screen genomes in this study, as well as parvovirus, bornavirus, and filovirus protein databases for further use (other commonly endogenized viruses). GTF files of annotated ERVs within the 19 Australian marsupial genomes and three American marsupial genomes are also available in this repository. Representative annotated ERV sequences from each of the eight novel lineages are available in a supplementary file in GenBank format. All marsupial genome assembles used are publicly available on NCBI, DNA Zoo, or Oz Mammals Genomics.
