# Supplementary material for: Invasion and Amplification of Endogenous Retroviruses in Dasyuridae Marsupial Genomes
Source: Mol Biol Evol. 2024 Aug 5;41(8):msae160. doi: 10.1093/molbev/msae160 (PMC11334065; doi:10.1093/molbev/msae160)
Supplement: msae160_Supplementary_Data [file msae160_supplementary_data.zip › Supplementary Figures.pdf]

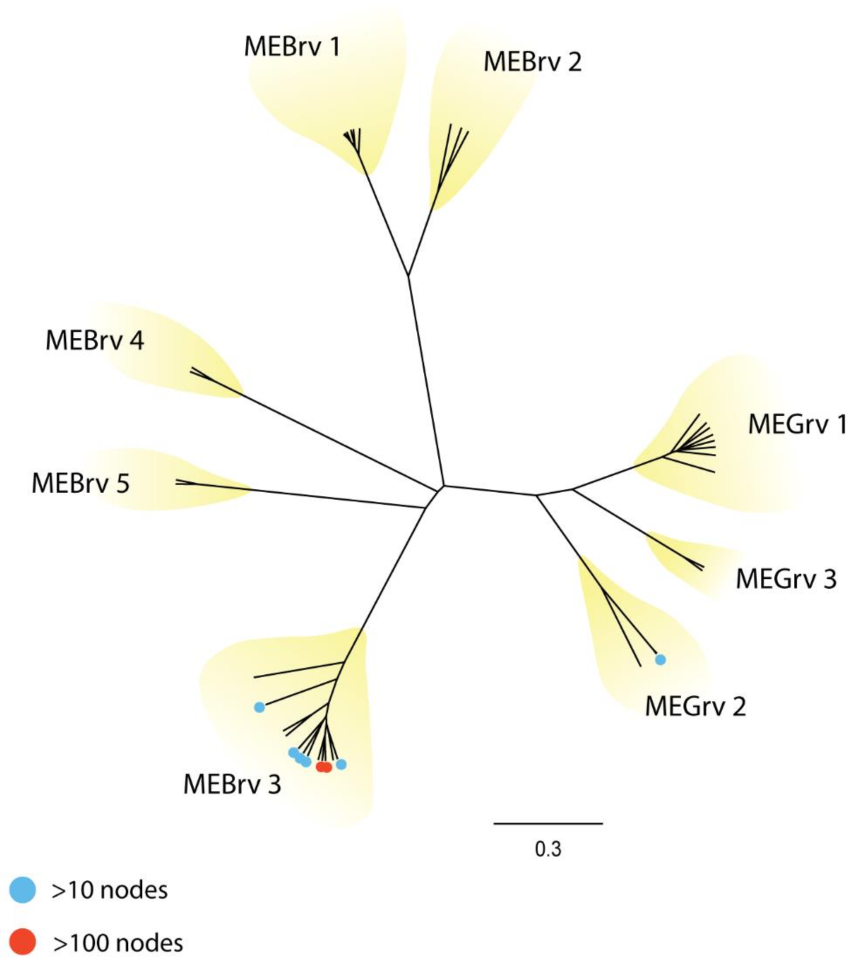

Supplementary Figure 1: Phylogenetic clustering of the 39 ERVs >6 kB within the Tasmanian devil genome.

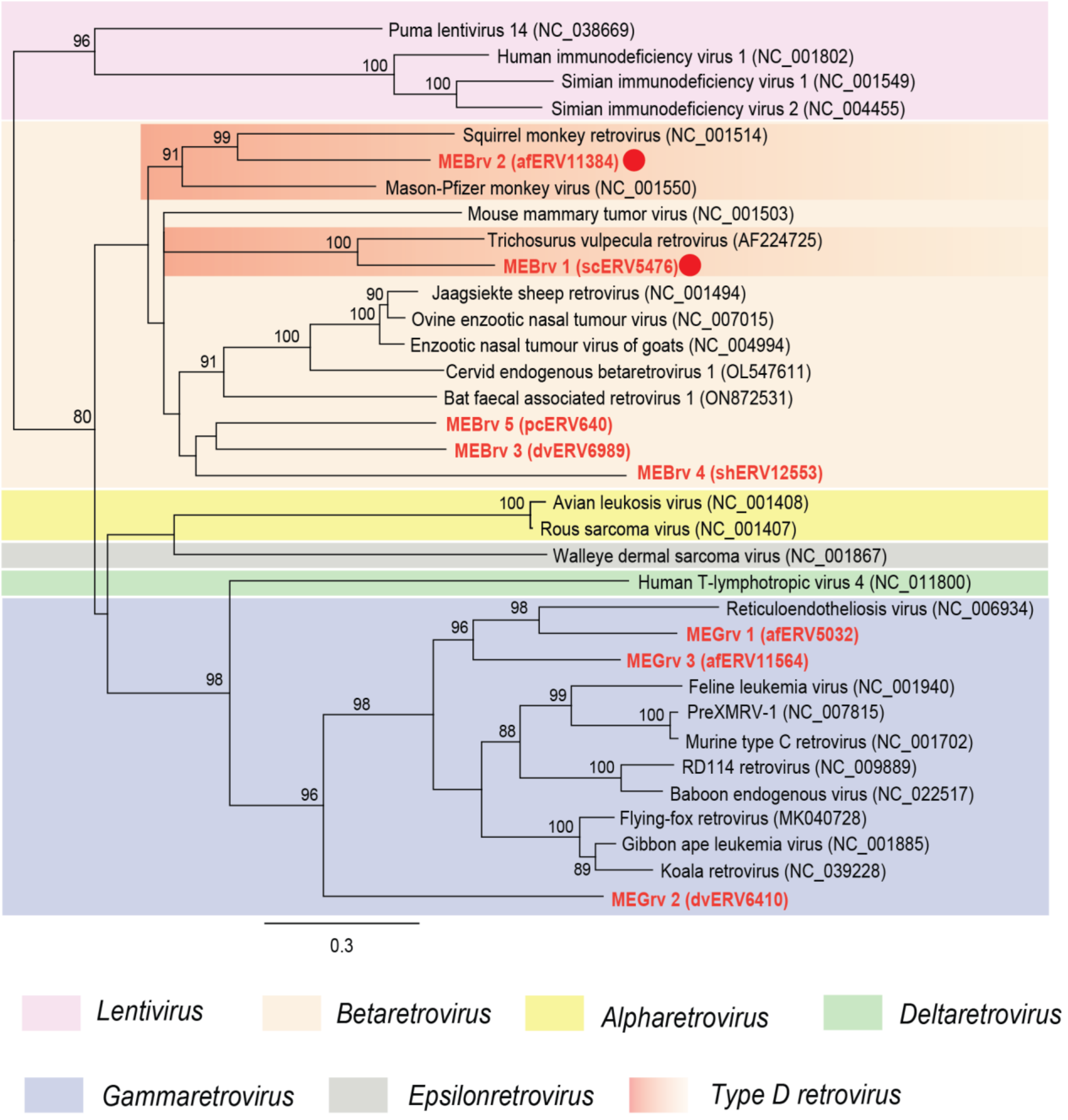

Supplementary Figure 2: Phylogeny of the gag region (608 nt) of marsupial retrovirus lineages compared to extant genera.
